# Amino acids to prevent cardiac surgery-associated acute kidney injury: a randomized controlled trial

**DOI:** 10.1186/s40981-024-00703-6

**Published:** 2024-03-26

**Authors:** Masahiro Kazawa, Daijiro Kabata, Hisako Yoshida, Kimito Minami, Takuma Maeda, Kenji Yoshitani, Hitoshi Matsuda, Ayumi Shintani

**Affiliations:** 1 Department of Critical Care Medicine, National Cerebral and Cardiovascular Center, 6-1, Kishibe-Shinmachi, Suita, Osaka Japan; 2 Department of Medical Statistics, Graduate School of Medicine, Osaka Metropolitan University, Osaka, Japan; 3 Department of Cardiovascular Surgery, National Cerebral and Cardiovascular Center, Osaka, Japan; 4 Department of Anesthesiology, National Cerebral and Cardiovascular Center, Osaka, Japan

**Keywords:** Acute kidney injury, Amino acid, Aortic surgery

## Abstract

**Background:**

This study aimed to examine the preventive effect of amino acids on postoperative acute kidney injury (AKI).

**Methods:**

This was single-center, patient- and assessor-blinded, randomized controlled trial. Patients who underwent aortic surgery with cardiopulmonary bypass were included. The intervention group received 60 g/day of amino acids for up to 3 days. The control group received standard care. The primary outcome was the incidence of AKI. We assessed the effect of amino acids on AKI using a Cox proportional hazards regression model.

**Results:**

Sixty-six patients were randomly assigned to the control or intervention group. One patient in the control group withdrew consent after randomization. The incidence of AKI was 10 patients (30.3%) in the intervention group versus 18 patients (56.2%) in the control group (adjusted hazard ratio, 0.44; 95% confidence interval, 0.20–0.95; *P* = 0.04).

**Conclusions:**

This trial demonstrated a significant reduction in AKI incidence with amino acid supplementation.

**Trial registration:**

jRCT, jRCTs051210154. Registered 31 December 2021, https://jrct.niph.go.jp/re/reports/detail/69916

**Supplementary Information:**

The online version contains supplementary material available at 10.1186/s40981-024-00703-6.

## Introduction

Acute kidney injury (AKI) is one of the most common complications after cardiovascular surgery, with an incidence of > 40% [1]. AKI is associated with a tenfold increase in mortality [2]. However, effective strategies for preventing AKI remain limited, and there is a strong demand for more efficacious preventative measures.

Previous studies have indicated that high-protein diets and amino acids can lead to reductions in renal vascular resistance, increases in renal blood flow, and improvements in estimated glomerular filtration rate (eGFR) by up to 35% [3–6]. Consequently, investigations have been conducted to explore whether amino acids have the potential to prevent AKI; however, a conclusive determination remains elusive. A previous study in which critically ill patients received intravenous amino acids reported no significant difference in the duration of renal dysfunction [7]. In contrast, Pu et al. demonstrated the potential benefits of amino acid administration in reducing AKI duration and improving eGFR in patients who underwent valve surgery and coronary artery bypass grafting [8]. However, the trial excluded patients who had undergone aortic surgery, a procedure with a high risk of inducing AKI. Therefore, we hypothesized that perioperative administration of amino acids might mitigate the occurrence of AKI, particularly after aortic surgery with cardiopulmonary bypass.

## Methods

### Ethical statement

This trial was conducted in compliance with the Declaration of Helsinki and Japanese laws regarding clinical research and complied with the guidelines prescribed by the Consolidated Standards of Reporting Trials statement. The trial was approved by the certified review board of the National Cerebral and Cardiovascular Center on 27 December 2021 (registration number: CRB2104) and registered with the Japan Registry of Clinical Trials (registration number: jRCTs051210154). All study participants provided written informed consent prior to enrollment in this trial.

### Study design

This study was a single-center, patient- and assessor-blinded, randomized controlled trial.

The institution in which the study was performed is a national center in Japan that specializes in cerebral and cardiovascular diseases.

### Eligibility criteria

Study participants must have met all of the following inclusion criteria:

1. able to undergo thoracic aortic replacement or thoracoabdominal aortic replacement;
2. > 20 years of age at the time of consent;
3. planned admission to the intensive care unit (ICU) after surgery;
4. provided informed consent.

Patients fulfilling one or more of the following criteria were excluded:

1. hemodialysis requirement;
2. preoperative eGFR < 15 mL/min/1.73 m^2^;
3. contraindication to receive Amiparen® (Otsuka Pharmaceutical Factory, Inc., Naruto, Tokushima, Japan);
4. pregnancy or lactation;
5. emergency surgery;
6. inappropriate for study inclusion in accordance with the research physician’s judgment.

### Anesthetic and postoperative management

In all patients, general anesthesia was performed with concomitant electrocardiography, pulse oximetry, non-invasive blood pressure measurement, and capnometry. Anesthesia was induced with midazolam, fentanyl, and rocuronium, and maintained with propofol, remifentanil, and rocuronium. After induction of general anesthesia, an arterial cannula was inserted in the radial artery, and a central venous catheter and a pulmonary artery catheter were inserted into the internal jugular vein. Continuous monitoring was implemented for arterial pressure, central venous pressure, pulmonary arterial pressure, and transesophageal echocardiography. Throughout the surgical procedure, dedicated anesthetists managed the patient’s condition. After the surgical procedure, the patient was transitioned to the ICU while being maintained under sedation. Subsequently, upon stabilization of their condition, the patient was roused from sedation and extubated following a successful ventilator withdrawal test. A water swallowing test was performed 6 h post-extubation, and the resumption of oral food intake commenced upon a successful result. In cases where extubation could not be accomplished within 48 h, the patient’s nutritional needs were met through enteral or intravenous feeding. Postoperative patient management was primarily by the attending surgeon.

### Interventions

Both the intervention and control groups received perioperative management from the attending physician. In the intervention group, Amiparen® (Otsuka Pharmaceutical Factory, Inc.), which is a comprehensive amino acid preparation, was administered intravenously at a dose of 200 mL (20 g) three times a day every 8 h up to 3 days (Supplementary Table S1 lists the levels of each amino acid in the solution). The first injection was given after the start of general anesthesia. The amino acid infusion was discontinued when the patient was started on an oral diet, enteral nutrition, or intravenous nutrition, or discharged from the ICU. In the control group, administration of Amiparen® was prohibited for 3 days after surgery. Aside from this distinction, the perioperative management protocols of the two groups were consistent.

### Outcomes

The primary outcome was AKI, which was defined in accordance with the Kidney Disease Improving Global Outcomes (KDIGO) criteria, as follows: an increase in serum creatinine of ≥ 0.3 mg/dL within 48 h; an increase in serum creatinine to ≥ 1.5 times the baseline level that was known or presumed to have occurred within the previous 7 days; or urine volume < 0.5 mL/kg/h for 6 h [9]. Additionally, creatinine (Cre)-AKI was defined as AKI diagnosed solely based on the creatinine levels, in accordance with the KDIGO criteria. Furthermore, the Cre-AKI stage was defined as follows, in accordance with the KDIGO criteria: stage 2, 2.0–2.9 times the baseline level; and stage 3, 3.0 times the baseline level, increase in serum creatinine to ≥ 4.0 mg/dl, or initiation of renal replacement therapy (RRT). Baseline creatinine was defined as the creatinine value closest to the date of surgery among patients tested up to 30 days before the date of surgery. Secondary outcomes were as follows: Cre-AKI, Cre-AKI stage, eGFR, urine output, discharge from the ICU, discharge from hospital, weaning from mechanical ventilation, initiation of RRT, and mortality rates. Blood urea nitrogen, aspartate aminotransferase, alanine aminotransferase, and total bilirubin, which were listed as adverse effects of the study drug in the manufacturer’s drug information, were chosen as safety outcomes in this study.

### Data collection, registration, assignment of intervention, and blinding

In this trial, we used the research electronic data capture software, REDCap® (Vanderbilt University Medical Center, Nashville, TN, USA) to record and store the data collected from each participant’s medical records. Eligible patients were identified from the list of patients for a scheduled surgery. Upon obtaining informed consent, patients were registered as research participants through REDCap®. We accessed each patient’s eligibility for enrollment in the study using the electronic case report form on REDCap®. Subsequently, a unique identification number was assigned. The allocation process was completed before the day before surgery. Outcomes were evaluated and recorded up to 3 months after surgery, regardless of whether patients were discharged from the hospital. The process of enrollment, allocation, outcome evaluation, and data entry were performed by research investigators independently of the attending surgeon.

Randomization was used to assign participants to either group in a 1:1 ratio. To evenly distribute the number of participants assigned to each group, the permuted block method (block size: 2 and 4) was used. Stratification was performed to control for any imbalances in the participants’ characteristics for the following factors: age (≥ 70 years or < 70 years), preoperative eGFR (≥ 60 mL/min/1.73 m^2^ or < 60 mL/min/1.73 m^2^), and type of surgery (thoracic aortic replacement or thoracoabdominal aortic replacement). A statistician generated the randomization sequence, and the REDCap® system assigned the patients to their respective groups.

The allocation of study drugs was concealed from the patients. To ensure blinding, the infusion bottle containing the crystalloid solution or the study drug was covered during administration. This prevented the participants from discerning the intervention that they were receiving. No placebo drugs were used. The trial was also assessor-blinded because AKI was determined by applying the KDIGO criteria based on serum creatinine and urine output.

### Sample size

In accordance with a previous report [10], the incidence of AKI, which was the primary outcome, was estimated as 70% in the control group. On the basis of data from Pu et al. [8], the relative risk reduction associated with amino acid administration was estimated as 50%, resulting in an assumed AKI rate of 35% in the intervention group. With this information, we performed a Monte Carlo simulation to determine the minimum number of participants required to achieve statistical power of 80% at a two-sided significance level of 5%. The estimated minimum number of participants was 30 in each group. Assuming a dropout rate of 10%, the enrollment target was set at 33 patients for each group.

### Statistical analysis

We estimated the cumulative probability of the primary outcome, AKI, using the Kaplan–Meier product-limit estimator. Furthermore, we assessed the effect of the amino acid infusion on postoperative AKI using a Cox proportional hazards regression model. In this analysis, patients were censored at the time of ICU discharge or 7 days after ICU admission, whichever occurred first, because monitoring urine output beyond ICU discharge was not feasible. The follow-up duration for AKI was 7 days, by definition.

To evaluate the effect of the amino acid infusion on the secondary outcomes, we performed a Cox proportional hazards regression analysis for Cre-AKI, discharge from the ICU, discharge from the hospital, weaning from mechanical ventilation, and initiation of RRT. The follow-up period for Cre-AKI was 7 days, and censoring was applied at 90 days for all other outcomes. For the Cre-AKI stage, an ordered logistic analysis was performed. Additionally, linear regression analysis was performed for eGFR and urine output. The normality of the residuals of the linear regression was assessed graphically. As a sensitivity analysis, linear regression analysis was performed on the difference between baseline Cre and the maximum Cre during the first 7 days after surgery, using age, sex, baseline creatinine, type of surgery, operation time, and total fluid volume from day 0 to day 2 as covariates.

The largest group of participants, apart from those who withdrew consent after allocation, was defined as the full analysis set. The per-protocol set was defined as the group excluding participants with serious violations of the study protocol. We performed the analyses described above using the full analysis set as a primary analysis followed by a sensitivity analysis using the per-protocol set. For the safety analysis, linear regression analysis was performed on blood testing data for the full analysis set. In all analyses, we adjusted for age, baseline eGFR, and type of surgery in the statistical models. This adjustment is recommended to improve the precision of the analysis, even when the baseline prognostic variables are balanced [11].

Continuous variables were summarized as median (interquartile range), while categorical variables and ordinal variables were summarized as number and percentage (%). All statistical analyses were performed using two-sided tests at a 5% significance level using R software, version 4.2.2 (www.r-project.org).

## Results

### Participants

**Fig. 1:**
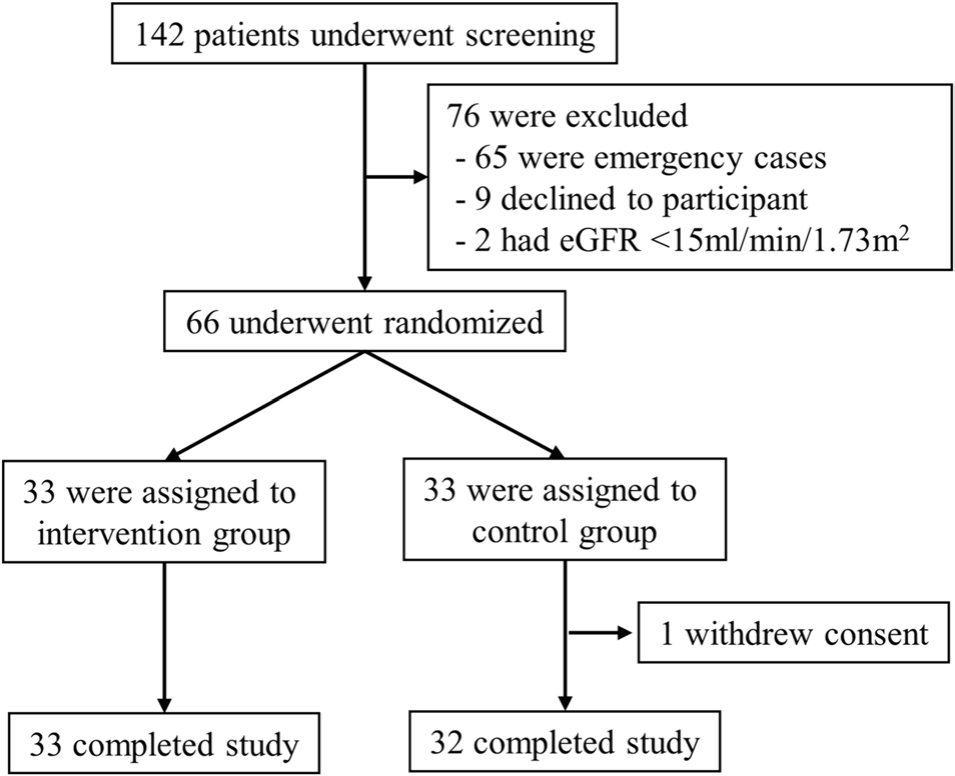
Consolidated Standards of Reporting Trials (CONSORT) 2010 patient recruitment flow diagram. eGFR, estimated glomerular filtration rate

Between February 2022 and January 2023, 142 patients were assessed for eligibility. Among them, 76 patients were excluded for various reasons, shown in the Consolidated Standards of Reporting Trials flow diagram (Fig. 1). Ultimately, 66 patients were randomly assigned in a 1:1 ratio to either the control or intervention group. However, one patient in the control group withdrew consent following allocation, resulting in final participant counts of 33 in the intervention group and 32 in the control group. Notably, one patient in the intervention group was non-compliant with the drug administration protocol. There were no discrepancies between the treatments assigned and administered. All participants included in the analysis completed the required follow-up period. The characteristics of the participants are summarized in Table 1. Age, baseline eGFR, and type of surgery were well-balanced between the two groups. The median (interquartile range) amount of amino acids administered with the study drug was 0.00 g (0.00, 0.00) in the control group and 46.67 g (40.00, 60.00) in the amino acid group. The total amino acids intake, encompassing those obtained from diet, enteral nutrition, and intravenous nutrition, and the study drug, were 0.00 g (0.00, 4.33) in the control group and 60.00 g (53.33, 60.00) in the amino acid group. Mean blood pressure and vasoactive-inotropic score [12], which means dopamine dose (mcg/kg/min) + dobutamine dose (mcg/kg/min) + 100 * noradrenaline dose (mcg/kg/min) during surgery did not differ between the two groups, however daily loop diuretic use was significantly higher in the control group (30.55 mg/day (5.32, 53.50) in the control group vs 5.20 mg/day (0.00, 25.50) in the intervention group; *P* = 0.011).

**Table 1:** Clinical and demographic characteristics of the study participants

**(a)**					
		**Control** **group**	**Intervention** **group**		
		*n* = 32	*n* = 33		
**Age (y)**		71 [56, 75]	72 [52, 77]		
**Sex, male**		65.6 (21)	48.5 (16)		
**Body Mass Index (kg/m**^**2**^**)**		21.3 [19.0, 25.2]	22.2 [20.4, 25.7]		
**Baseline eGFR (ml/min/1.73m**^**2**^**)**		64 [45, 80]	67 [56, 75]		
**Baseline creatinine (mg/dL)**		0.87 [0.70, 1.14]	0.79 [0.66, 0.93]		
**Type of surgery**					
TAAR		84.4 (27)	84.8 (28)		
TAAAR		15.6 (5)	15.2 (5)		
**Comorbidities**					
Hypertension		65.6 (21)	72.7 (24)		
Diabetes mellitus		9.4 (3)	12.1 (4)		
Dyslipidemia		25.0 (8)	33.3 (11)		
COPD		12.5 (4)	3.0 (1)		
History of thoracic surgery		28.1 (9)	30.3 (10)		
LVEF		61 [59, 65]	60 [60, 65]		
**(b)**					
	**Control** **group**	**Intervention** **group**		**SMD**	***P*** **value**
	*n* = 32	*n* = 33			
**Dosage of amino acids from study drug (g/day)**	0.0 [0.0, 0.0]	46.7 [40.0, 60.0]		7.045	< 0.001
**Total intake of amino acids** **(g/day)**	0.0 [0.0, 4.3]	60.00 [53.3, 60.0]		7.256	< 0.001
**Operation time (minutes)**	499 [424, 599]	439 [380, 578]		0.33	0.084
**CPB time (minutes)**	287 [227, 338]	242 [226, 275]		0.484	0.484
**Cross-clamp time (minutes)**	167 [111, 230]	134 [102, 182]		0.235	0.158
**Use of DHCA**	78.1 (25)	81.8 (27)		0.092	0.71
**DHCA time (minutes)**	45 [33, 64]	32 [27, 59]		0.22	0.389
**Maximum creatinine (mg/dL)**	1.25 [0.82, 1.83]	0.92 [0.83, 1.24]		0.617	0.079
**mBP during CPB (mmHg)**	56.50 [52.50, 62.25]	57.00 [53.00, 66.00]		0.094	0.563
**mBP post CPB (mmHg)**	69.00 [67.00, 74.25]	69.00 [65.00, 73.00]		0.069	0.679
**VIS**	3.03 [2.38, 4.62]	2.70 [2.03, 3.92]		0.077	0.273
**Loop diuretics (mg/day)**	30.55 [5.32, 53.50]	5.20 [0.00, 25.50]		0.704	0.011
**Total fluid volume (mL)**					
Day 0	11,286 [9266, 13450]	10,783 [8849, 15364]		0.049	0.665
Day 1	2929 [2346, 3798]	3517 [3039, 4556]		0.060	0.011
Day 2	1346 [966, 2452]	2150 [930, 2550]		0.048	0.498
**RCC (mL)**					
Day 0	3861 [3053, 5043]	3640 [2686, 5268]		0.537	0.067
Day 1	210 [0, 676]	50 [0, 268]		0.057	0.391
Day 2	0 [0, 0]	0 [0, 0]		0.302	0.356

Participant characteristics for the factors (a) before allocation and (b) after allocation. Medians and interquartile ranges are presented for continuous variables. Proportions (%) and frequencies are presented for categorical variables. The dosage of amino acids from the study drug and the total intake of amino acids are shown for the initial 3 days after surgery. The total intake of amino acids includes those obtained from the patient’s diet, enteral nutrition, and intravenous nutrition, as well as the study drug. DHCA times are data only for cases in which DHCA was used. Maximum creatinine means the maximum value up to 7 days after surgery. Loop diuretics are shown as mean daily doses up to 7 days after surgery. *P*-values were calculated using Student’s t test for continuous variables and the Chi-square test for binary variables

*COPD* chronic obstructive pulmonary disease, *CPB* cardiopulmonary bypass, *DHCA* deep hypothermic circulatory arrest, *eGFR* estimated glomerular filtration rate, *LVEF* left ventricular ejection fraction, *mBP* mean blood pressure, *RCC* red cell concentrate, *SMD* standardized mean difference, *TAAR* thoracic aortic replacement, *TAAAR* thoracoabdominal aortic replacement; total fluid volume, total volume of fluids and blood transfusions on each day, *VIS* vasoactive-inotropic score, which means dopamine dose (mcg/kg/min) + dobutamine dose (mcg/kg/min) + 100 * noradrenaline dose (mcg/kg/min), *y* year

### Outcomes

**Fig. 2:**
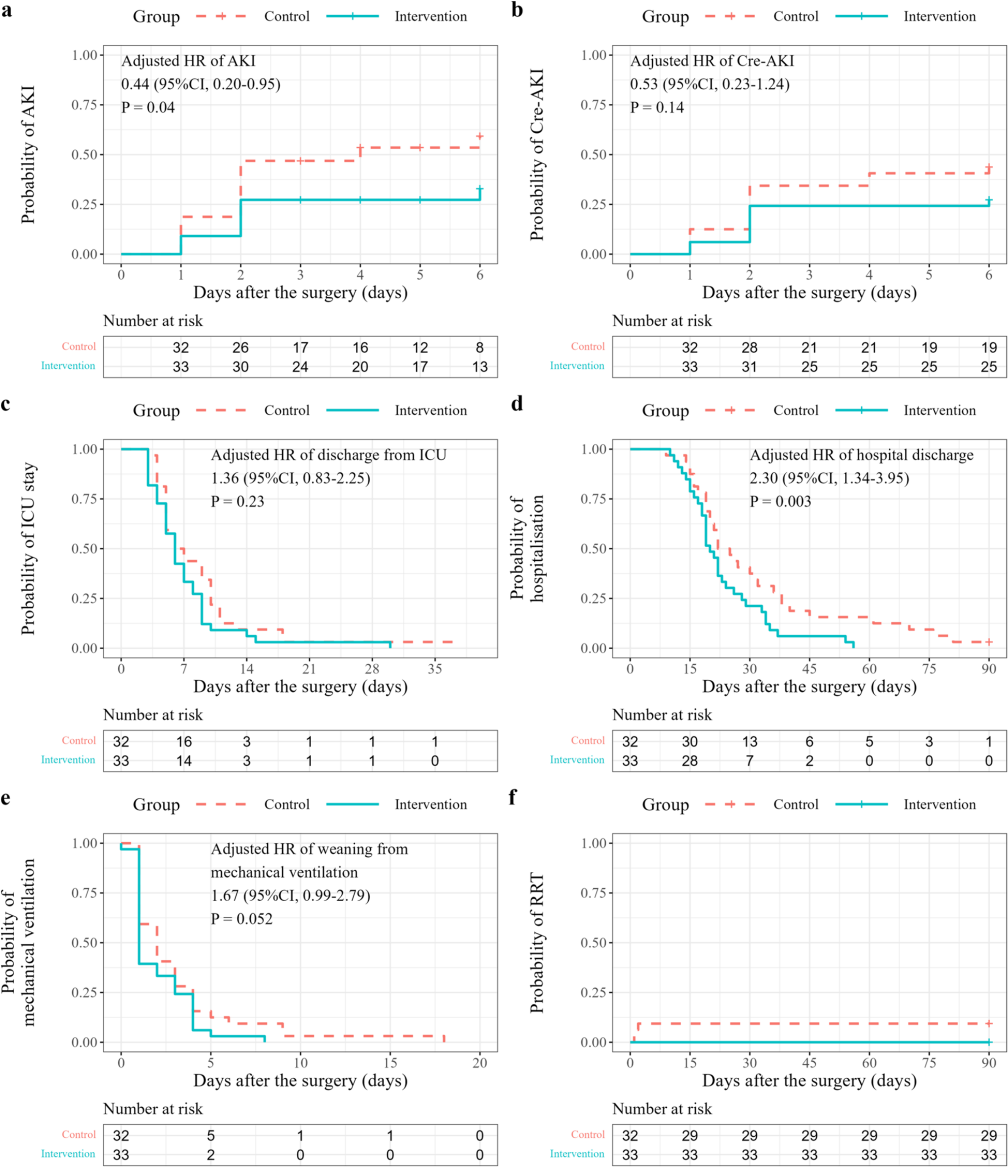
Kaplan–Meier curves for the outcomes. Kaplan–Meier curves are shown for the probability of **a** AKI, **b** Cre-AKI, **c** ICU stay, **d** hospitalization, **e** mechanical ventilation, and **f** RRT. Hazard ratios for **a** AKI, **b** Cre-AKI, (**c**) discharge from the ICU, **d** discharge from hospital, and **e** weaning from mechanical ventilation were obtained using Cox proportional hazards regression models. As RRT was administered to only three patients in the control group, no statistical analysis was performed for this variable. AKI, acute kidney injury; Cre-AKI, acute kidney injury defined only based on the creatinine value, in accordance with the Kidney Disease Improving Global Outcomes Criteria; CI, confidence interval; ICU, intensive care unit; RRT, renal replacement therapy

**Fig. 3:**
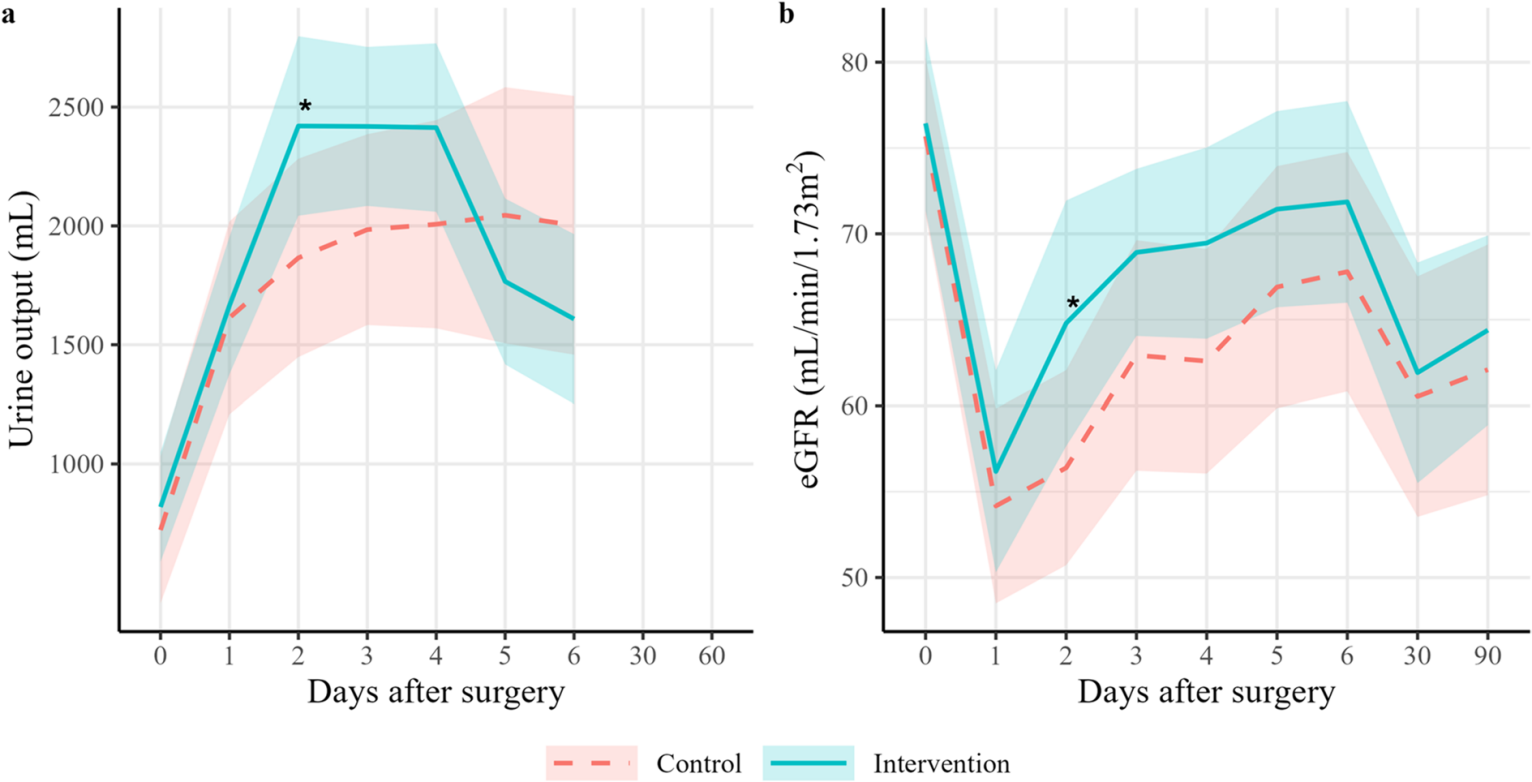
Trends in urine output and eGFR. Multivariable linear regression was performed to compare **a** urine output and **b**) eGFR between the treatment groups in the full analysis set. Shading indicates 95% confidence intervals. The asterisk indicates a statistically significant difference between the two groups (*P* < 0.05). eGFR, estimated glomerular filtration rate

The results for the full analysis set are reported in the main text, and those for the per-protocol set are provided in the Supplementary materials. There were no deaths in either group. AKI occurred in 18 patients (56.2%) in the control group vs. 10 patients (30.3%) in the intervention group (adjusted hazard ratio (HR), 0.44; 95% confidence interval (CI), 0.2–0.95; *P* = 0.04) (Fig. 2). Cre-AKI occurred in 14 patients (43.8%) in the control group vs. 9 patients (27.3%) in the intervention group (adjusted HR, 0.68; 95% CI, 0.28–1.62; *P* = 0.38). The number of Cre-AKI occurrences per stage in each group is shown in Table 2 (adjusted odds ratio, 0.39; 95% CI, 0.13–1.17; *P* = 0.09). Kaplan–Meier curves and analysis results for the secondary outcomes are shown in Fig. 2. The probability of discharge from hospital was significantly higher in the intervention group vs. the control group (adjusted HR, 2.30; 95% CI, 1.34–3.95; *P* = 0.003). As RRT was required for only three participants in the control group, no statistical analyses were performed for this variable. Figure 3 shows the trends in urine output and eGFR. The intervention group had significantly higher urine output on day 2 (control vs. intervention, respectively: 1865 mL (95% CI, 1448–2283) vs. 2420 mL (95% CI, 2043–2798) (*P* = 0.049). Similarly, the intervention group had significantly higher eGFR on day 2 (control vs. intervention, respectively: 56.4 mL/min/1.73 m^2^ (95% CI, 50.7–62.1) vs. 64.8 mL/min/1.73 m^2^ (95% CI, 57.6–71.9) (*P* = 0.049). Sensitivity analysis showed that the difference between baseline Cre and maximal Cre was significantly smaller in the intervention group (-0.30, 95%CI, -0.50—0.09, *P* = 0.006). Regarding the safety outcomes, blood urea nitrogen levels on day 2 were higher in the intervention group compared with the control group (control vs. intervention: 30.2 mg/dL (95% CI, 26.0–34.4) vs. 35.9 mg/dL (95% CI, 32.8–39.1) (*P* = 0.02) (Supplementary Figure S3).

**Table 2:** Incidence of AKI in each group

	**Control** **group**	**Intervention** **group**	**Effect**	***P*** **value**
	*n* = 32	*n* = 33		
**AKI**	56.2 (18)	30.3 (10)	HR 0.44 (0.20–0.95)	0.04
**Cre-AKI**	43.8 (14)	27.3 (9)	HR 0.53 (0.23–1.24)	0.14
**Cre-AKI stage**				
0	56.2 (18)	72.7 (24)	OR 0.39 (0.13–1.17)	0.09
1	31.2 (10)	24.2 (8)		
2	3.1 (1)	3.0 (1)		
3	9.4 (3)	0.0 (0)		

Proportions (%) and frequencies are presented for each variable. For the full analysis set, Cox proportional hazards regression analysis was performed for AKI and Cre-AKI, and ordered logistic regression analysis was performed for Cre-AKI stage. Numbers in parentheses in the Effect column indicate 95% confidence intervals

*AKI* acute kidney injury, *Cre-AKI* acute kidney injury defined only on the basis of the creatinine value, in accordance with the Kidney Disease Improving Global Outcomes Criteria, Cre-AKI stage, staged *Cre-AKI*, *HR* hazard ratio, *OR* odds ratio

## Discussion

This study was a randomized controlled trial to examine whether perioperative amino acids reduce the incidence of AKI after aortic surgery with cardiopulmonary bypass. The trial demonstrated a remarkable reduction in the incidence of AKI within the intervention group. The control group had an AKI incidence rate of 56.2%, while the intervention group had a significantly lower rate of 30.3%. The statistical analysis further confirmed the efficacy of the intervention, revealing a significant reduction in AKI incidence (adjusted HR, 0.44; 95% CI, 0.20–0.95; *P* = 0.04).

Although the pilot nature and the limited number of participants prevented the achievement of statistical significance, in a randomized controlled trial by Pu et al.[8], the incidence of AKI was 20% in the control group and 9% in the intervention group, indicating a comparable risk ratio with our results. Thus, the findings of the present trial support and align with those of previous research in this field. Moreover, the dosage of amino acids in the previous study was 100 g/day. However, the dosage was reduced to 60 g/day in the present trial. This was performed because the average participant in the previous study weighed more than 80 kg, while the average participant in the present study weighed approximately 65 kg, and the recommended daily protein intake by the Japanese Ministry of Health, Labour and Welfare was 60 g/day.

Several mechanisms have been hypothesized as potential mechanisms by which amino acid administration may affect renal function. One report suggested that amino acids are filtered by the glomerulus, thereby enhancing proximal tubular absorption and increasing GFR. Another report suggested that amino acids cause local nitric oxide and prostaglandin release, resulting in vasodilatation and increasing renal blood flow and GFR [13]. In fact, urine output on day 2 in this trial was significantly higher in the intervention group than that in the control group. This suggests that the administration of amino acids increased urine volume by increasing renal blood flow. This mechanistic insight provides a plausible explanation for the observed renoprotective effects of amino acid administration in this trial. These findings highlight the potential clinical significance of amino acid administration as a strategy to improve renal outcomes after cardiovascular surgery. Further research is necessary to reveal the specific underlying mechanisms and optimize the dosage and duration of amino acid administration for maximum therapeutic benefit. Nonetheless, this trial contributes valuable insights into the potential of amino acids in renal protection and opens avenues for future investigations in this field.

The strength of this trial is its rigorous adherence to the KDIGO criteria for diagnosing AKI. Notably, only a few studies of AKI incorporated urine volume criteria into the diagnosis. However, in this study, we included urine volume as a factor in determining AKI. One challenge that we encountered was the inability to measure urine output after discharge from the ICU. To overcome this limitation, we performed Cox proportional hazards regression analysis with ICU discharge as the censoring event. This close adherence to internationally recognized criteria enabled a standardized and reliable diagnosis.

Conversely, there was no statistically significant difference in Cre-AKI and Cre-AKI stage between the two groups. This implies that the difference in AKI incidence between the groups may stem from the criterion related to urine output. This observation is not contradictory, given that amino acid infusion is known to augment renal blood flow and subsequently increase urine output. However, there remains a debate regarding whether this effect truly suppresses AKI. This is because the effect appears to primarily enhance urine output, potentially allowing the diagnosis of AKI to escape detection by standard AKI criteria, similar to the action of diuretics, generally. Similarly, no significant differences in eGFR were found at the 3-month follow-up. Therefore, it appears that eGFR levels might recover long term even without the use of amino acids. This also suggests that amino acids may not have a substantial effect on renal dysfunction. Notably, we did not measure renal injury biomarkers, such as neutrophil gelatinase-associated lipocalin because the hypothesis of this study was that amino acids suppress AKI diagnosed in accordance with the KDIGO criteria. Therefore, it may be necessary to measure such biomarkers to confirm direct renal tubular injury.

This trial has several limitations. First, this was a single-center study performed in a national center specialized in cerebral and cardiovascular diseases, which may limit the generalizability of the findings. To address this, standard care was administered outside of the intervention, and the incidence of AKI in the control group was comparable to that in previous studies. Additionally, we sought to mitigate the impact of the single-center design by applying the KDIGO criteria, which are recognized for their simplicity and widespread use in diagnosing AKI. Second, the group allocations were not blinded to the healthcare providers and researchers, and no placebo medication was administered to the control group. This was because we followed the design of a previous study by Pu et al. [8]. These issues could have affected the outcomes and introduced potential bias. However, serum creatinine measurements were performed objectively by a central laboratory unaware of the treatment allocation, and the diagnosis of AKI was established by rigorously applying the KDIGO criteria. Therefore, the lack of blinding and the absence of a placebo control had a relatively modest impact on the results.

In conclusion, this trial highlighted a significant reduction in AKI incidence achieved through the perioperative administration of amino acids. Further research should involve a multicenter, placebo-controlled, double-blind, randomized controlled trial, which would provide stronger evidence and enhance the generalizability of this study’s findings.

### Supplementary Information

**Supplementary Material 1.**

## Data Availability

The datasets used and/or analyzed during the current study are available from the corresponding author on reasonable request.
