# Supplementary material for: Amino acids to prevent cardiac surgery-associated acute kidney injury: a randomized controlled trial
Source: JA Clin Rep. 2024 Mar 26;10:19. doi: 10.1186/s40981-024-00703-6 (PMC10963352; doi:10.1186/s40981-024-00703-6)
Supplement: Supplementary file 1 — Supplementary Material 1. [file 40981_2024_703_MOESM1_ESM.docx]

**Supplementary Table S1: Content of the Mixed Amino Acid Solution (g/200ml)**

| L‐Leucine | 2.8 |
| --- | --- |
| L‐Isoleucine | 1.6 |
| L‐Valine | 1.6 |
| L‐Lysine acetate | 2.96 |
| L‐Lysine | 2.1 |
| L‐Threonine | 1.14 |
| L‐Tryptophan | 0.4 |
| L‐Methionine | 0.78 |
| L‐Cysteine | 0.2 |
| L‐Phenylalanine | 1.4 |
| L‐Tyrosine | 0.1 |
| L‐Arginine | 2.1 |
| L‐Histidine | 1 |
| L‐Alanine | 1.6 |
| L‐Proline | 1 |
| L‐Serine | 0.6 |
| Glycine | 1.18 |
| L‐Aspartate | 0.2 |
| L‐Glutamate | 0.2 |

**Supplementary Table S2: Incidence of AKI in each group in the per-protocol set**

|  | **Control group** | **Intervention group** | **Effect** | **P value** |
| --- | --- | --- | --- | --- |
|  | n = 32 | n = 32 |  |  |
| **AKI** | 56.2 (18) | 28.1 (9) | HR 0.40 (0.18-0.89) | 0.02 |
| **Cre-AKI** | 43.8 (14) | 25.0 (8) | HR 0.48 (0.20-1.15) | 0.10 |
| **Cre-AKI stage** |  |  |  |  |
| 0 | 56.2 (18) | 75.0 (24) | OR 0.37 (0.12-1.11) | 0.08 |
| 1 | 31.2 (10) | 21.9 (7) |  |  |
| 2 | 3.1 (1) | 3.1 (1) |  |  |
| 3 | 9.4 (3) | 0.0 (0) |  |  |

Proportions (%) and frequencies are presented for each variable. For the per-protocol set, Cox proportional hazard regression analysis was performed for AKI and Cre-AKI, and ordered logistic regression analysis was performed for Cre-AKI stage. Numbers in parentheses in the Effect column indicate 95% confidence intervals. AKI, acute kidney injury; Cre-AKI, acute kidney injury defined based only on the basis of the creatinine value, in accordance with the Kidney Disease Improving Global Outcomes Criteria; Cre-AKI stage, staged Cre-AKI; HR, hazard ratio; OR, odds ratio.

**Supplementary Figure S1: Kaplan–Meier curves for the outcomes in the per-protocol set**

**
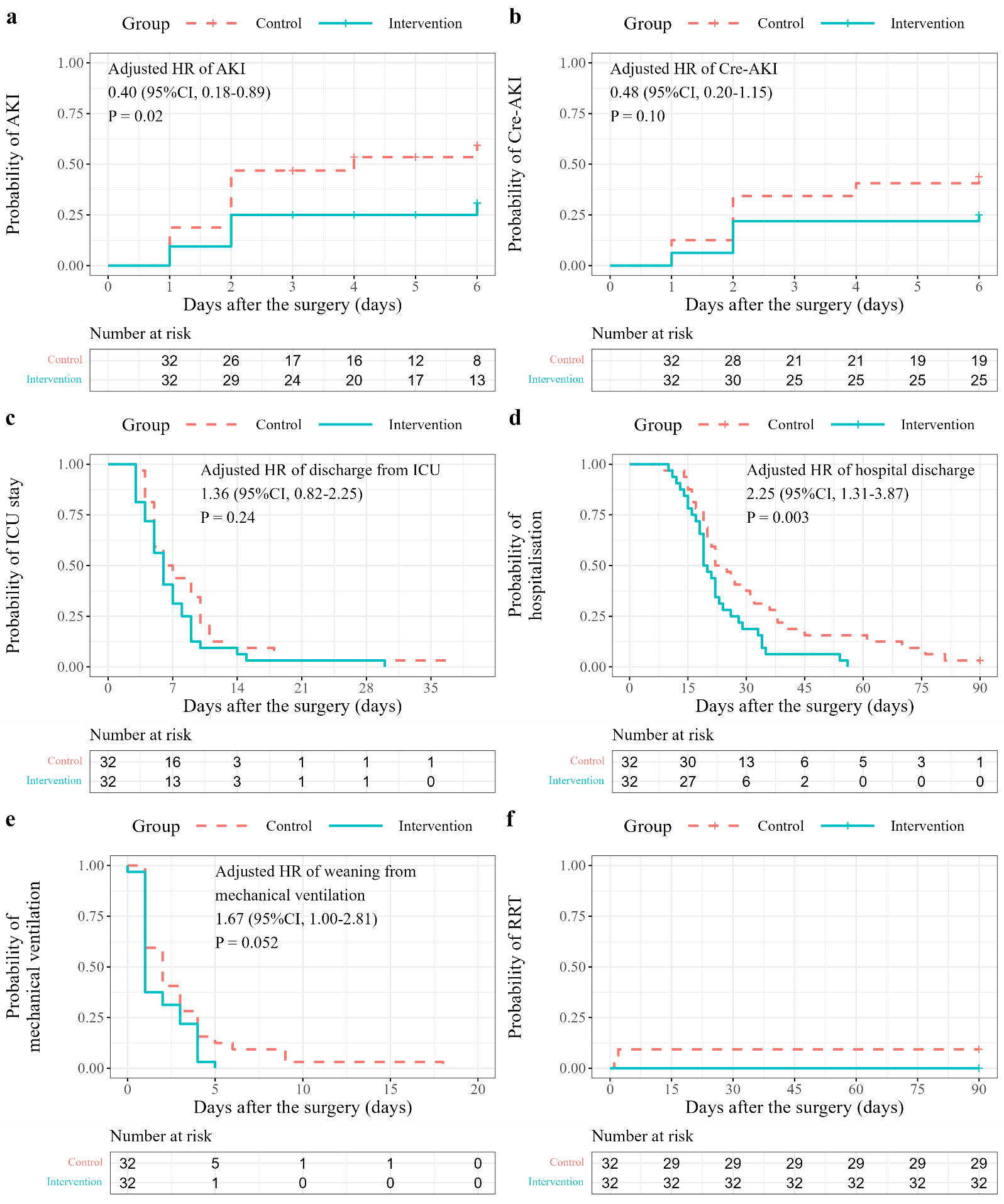
**

Kaplan–Meier curves are shown for the probability of (a) AKI, (b) Cre-AKI, (c) ICU stay, (d) hospitalization, (e) mechanical ventilation, and (f) RRT. Hazard ratios for (a) AKI, (b) Cre-AKI, (c) discharge from ICU, (d) discharge from hospital, and (e) weaning from mechanical ventilation were obtained using Cox proportional hazards regression models. As RRT was administered to only three patients in the control group, no statistical analysis was performed for this variable. AKI, acute kidney injury; Cre-AKI, acute kidney injury defined only on the basis of the creatinine value, in accordance with the Kidney Disease Improving Global Outcomes Criteria; CI, confidence interval; ICU, intensive care unit; RRT, renal replacement therapy.

**Supplementary Figure S2: Trends in urine output and eGFR in the per-protocol set**

**
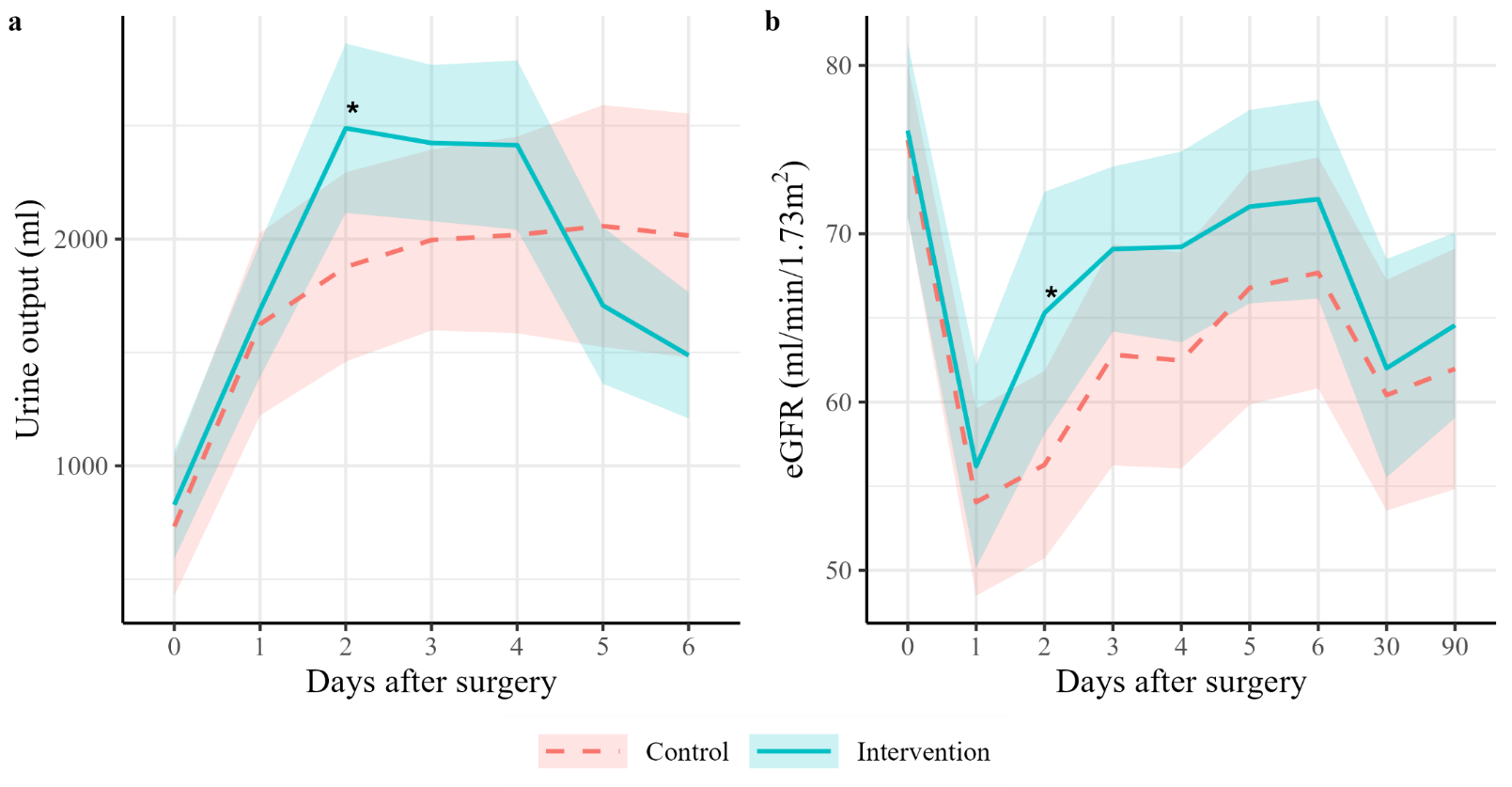
**

Multivariable linear regression was performed to compare (a) urine output and (b) eGFR between the treatment groups in the per-protocol set. Shading indicates 95% confidence intervals. The asterisk indicates a statistically significant difference between the two groups (P < 0.05).

eGFR, estimated glomerular filtration rate.

**Supplementary Figure S3: Trends in safety outcomes**

**
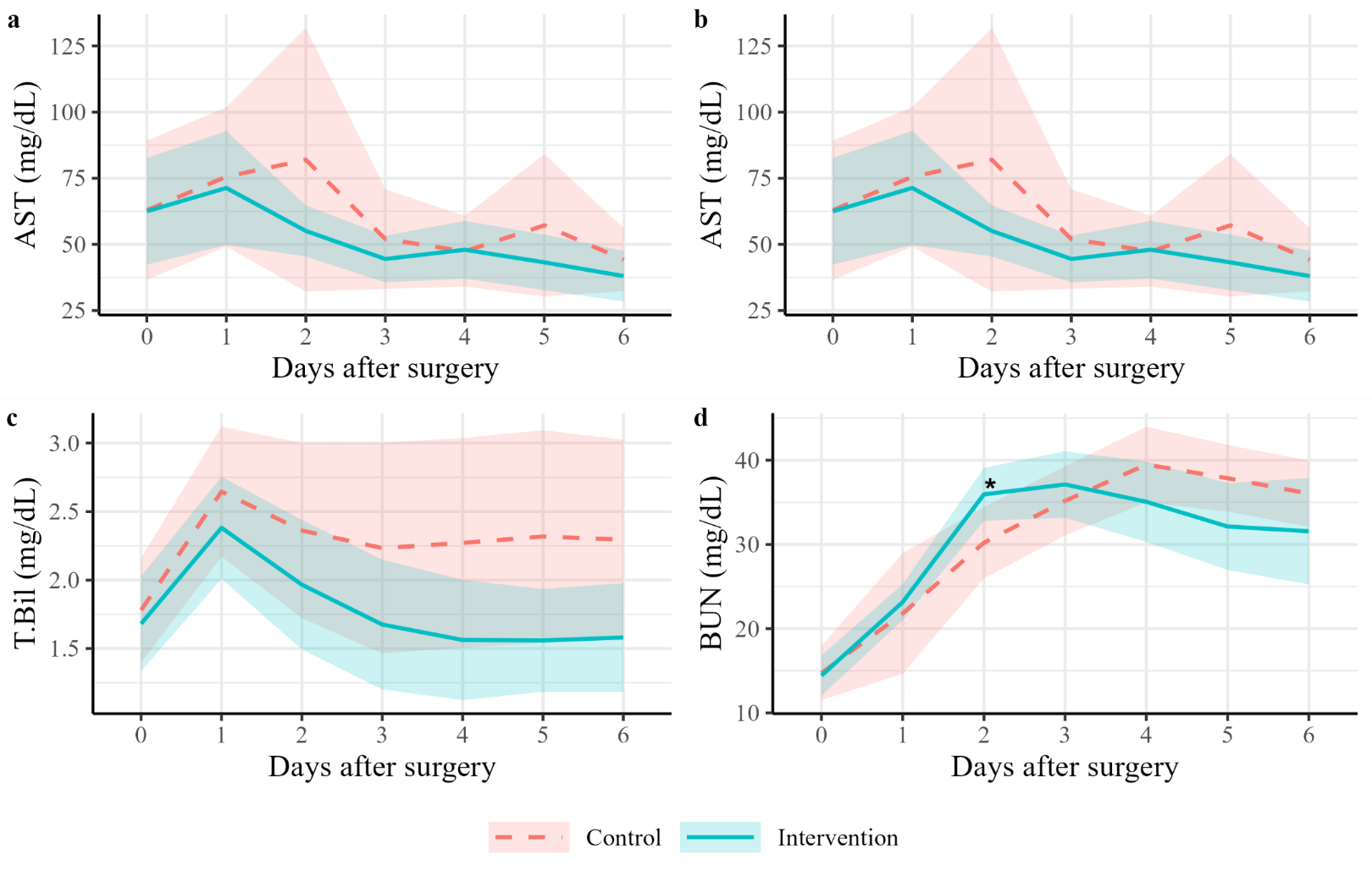
**

Multivariable linear regression was performed to compare (a) aspartate aminotransferase, (b) alanine aminotransferase, (c) total bilirubin, and (d) blood urea nitrogen levels between treatment groups. Shading indicates 95% confidence intervals. The asterisk indicates a statistically significant difference between the two groups (P<0.05).

BUN, blood urea nitrogen; ALT alanine aminotransferase; AST, aspartate aminotransferase; T. Bil, total bilirubin.
